# Human Amniotic Fluid Mesenchymal Stem Cell-Derived Exosomes Inhibit Apoptosis in Ovarian Granulosa Cell via miR-369-3p/YAF2/PDCD5/p53 Pathway

**DOI:** 10.1155/2022/3695848

**Published:** 2022-07-26

**Authors:** Zixiang Geng, Haiyang Chen, Gang Zou, Long Yuan, Peng Liu, Bingrong Li, Kaiyong Zhang, Fangyuan Jing, Xiaoli Nie, Te Liu, Bimeng Zhang

**Affiliations:** ^1^Department of Acupuncture and Moxibustion, Shanghai General Hospital, Shanghai Jiao Tong University School of Medicine, Shanghai 200086, China; ^2^Shuguang Hospital, Shanghai University of Traditional Chinese Medicine, Shanghai 201203, China; ^3^Shanghai Geriatric Institute of Chinese Medicine, Shanghai University of Traditional Chinese Medicine, Shanghai 200031, China; ^4^Department of Obstetrics, Shanghai First Maternity and Infant Hospital, Tongji University School of Medicine, Shanghai 200040, China

## Abstract

Human amniotic fluid stem cell-derived exosome (HuAFSC-exosome) transplantation is considered a promising treatment for premature ovarian failure (POF). However, its mechanism remains unclear. In this study, exosomes were isolated and enriched from HuAFSC subsets of CD44+/CD105+, and the exosomes were transplanted into a POF model *in vitro* and *in vivo*. Our results confirmed that the exosomes produced by CD44+/CD105+ HuAFSCs could achieve therapeutic effects in a mouse POF model. Our research also showed that CD44+/CD105+ HuAFSC-exosomes carrying miR-369-3p could specifically downregulate the expression of YAF2, inhibit the stability of PDCD5/p53, and reduce the apoptosis of ovarian granulosa cells (OGCs), thereby exerting therapeutic effects on POF. Knowledge of these mechanisms demonstrates that miRNAs carried by CD44+/CD105+ HuAFSC-exosomes are critical to the therapy of POF. This will be useful for the clinical application of stem cells.

## 1. Introduction

Premature ovarian failure (POF) is a disease characterized by a lack of mature follicles in women before the age of 40 years, accompanied by decreased estrogen levels and elevated gonadotropin levels [1, 2]. The common clinical manifestations of POF are hot flashes and sweating, insomnia, amenorrhea, and sexual dysfunction, which seriously affect women's fertility and physical and mental health [3, 4]. The occurrence of POF is closely related to the quality and state of ovarian granulosa cells (OGCs) [5]. In general, the decline in ovarian reserve function is thought to be the premise of premature ovarian failure, while the senescence and apoptosis of OGCs are considered to be the reasons for the decrease in ovarian reserve [6, 7]. At present, hormone replacement therapy is the main treatment for POF, but it is accompanied by serious side effects [8–10]. Therefore, a safer treatment with fewer side effects is needed.

Amniotic fluid mesenchymal stem cells (AFSCs) are a group of stem cells that reside within the amniotic fluid of pregnant women. They express mesenchymal stem cell markers, such as CD44, CD29, CD90, and CD105, have low immunogenicity, and can be induced to undergo pluripotent differentiation into a variety of tri-germ cells under specific conditions [11–14]. Moreover, our previous studies found that CD44+/CD105+ AFSCs could survive for a long time in POF mice and expressed a variety of growth factors, such as epidermal growth factor, basic fibroblast growth factor, transforming growth factor *α β*, and bone morphogenetic protein 4 [11, 15]. Moreover, the abovementioned cells can significantly repair the ovarian tissue of POF mice [12]. However, the mechanism by which these cells repair ovarian tissue in POF mice remains unclear. It is established that mesenchymal stem cells can continuously release exosomes, which can play a role in cell therapy [16–18]. Exosomal vesicles are small 40–150 nm particles from *in vivo* sources that carry genetic material, such as mRNA, miRNA, and bioactive proteins, to target organs [19–21]. Exosomes also play a key role in the paracrine signal transduction of stem cells and intercellular communication [22, 23]. In addition, exosomes show regeneration-promoting properties similar to stem cells, and therefore, direct treatment with exosomes can avoid many of the adverse effects of stem cell transplantation [24]. Previous studies have shown that exosomes derived from bone marrow mesenchymal stem cells could achieve therapeutic effects in a POF animal model [25, 26].

MicroRNA (miRNA) is a special type of RNA, of ~20–23 bp, that does not encode protein [27, 28]. miRNA can recognize and bind to a specific sequence in the target gene, induce AGO-mediated cleavage, and silence the expression of the target gene [1]. miRNA is an important transcription factor *in vivo*, widely involved in cell proliferation, apoptosis and differentiation, tissue development and metabolism, and other life processes [29]. Our studies have confirmed that miR-15a, miR-15b, and miR-146b-5p could affect the occurrence and development of POF in several ways [1, 5, 30]. There is therefore a close relationship between miRNA and POF.

YAF2 is the binding molecule of Yin-Yang-1 (YY-1) protein and plays an important role in transcriptional regulation [31]. In recent studies, YAF2 has been shown to stabilize the structure of PDCD5 and promote the expression of p53 [32]. PDCD5 protein is an apoptosis-related protein that is widely expressed in different tissues. It plays an important positive regulatory role in the process of apoptosis and can participate in apoptosis by activating stable caspase-3, p53, HIF-1*α*, and other pathways [33–35]. However, the regulatory role of YAF2 and PDCD5 in POF has not been reported in detail.

In this study, we explored the efficacy of CD44+/CD105+ AFSC-derived exosomes in the treatment of POF. We also confirmed the hypothesis that the exosomes of CD44+/CD105+ AFSCs carried miR-369-3p that regulated YAF2 expression and disrupted the stability of PDCD5/p53, thus inhibiting the apoptosis of mouse OGCs.

## 2. Materials and Methods

### 2.1. Isolation and Culture of Mouse Ovarian Granulosa Cells (OGCs)

According to previous studies [5, 6, 36], 7-week-old female C57BL/6 mice were purchased from the Experimental Animal Center of Shanghai University of Traditional Chinese Medicine. The mice were euthanized by cervical dislocation. Ovarian tissue was isolated under aseptic conditions and placed in 4°C phosphate-buffered saline (PBS). The tissue was then cut into pieces, and 2 mL of hyaluronidase (0.1%, Sigma-Aldrich, St. Louis, MO, USA) was added and incubated at 37°C for 1 min to digest the tissue. The tissue suspension was gently blown, and 200 *μ*L of fetal bovine serum (Gibco, Gaithersburg, MD, USA) was added to stop the digestion. The suspension was then filtered through a 200-mesh cell filter, and the filtrate was mixed with 5 mL of PBS and centrifuged at 1,500 rpm at 10°C for 5 min. The precipitate was resuspended in 5 mL of PBS and centrifuged at 1,500 rpm at 10°C for 5 min. The pellet was resuspended in Dulbecco's modified Eagle's medium: Ham's Fmur12 medium (DMEM: F12) (1 : 1). The culture medium contained 10% fetal bovine serum, 10 ng/mL basic fibroblast growth factor (bFGF), 10 ng/mL epidermal growth factor (EGF), 2 ng/mL glutamine, 10 ng/mL growth hormone (Gh), and 15 ng/mL estradiol (E2) (Gibco). The cell suspension was inoculated into a six-well cell culture plate and then incubated at 37°C and 5% CO_2_ until 80% confluence.

### 2.2. Establishment of the POF Mouse Model and Intervention with Exosomes

Based on our previous study [5, 6, 36], 36 10-week-old female C57BL/6 mice were randomly divided into three groups (WT group, POF+ saline group, POF+ exosome group) with 12 rats in each group. POF mice were first intraperitoneally injected with cyclophosphamide (Sigma-Aldrich, St. Louis, MO, USA) at 70 mg/kg for 1 week, and then, cyclophosphamide was injected intraperitoneally at a dose of 30 mg/kg for 2 weeks every 2 days to establish the POF mouse model. In addition, the mice in the control group were intraperitoneally injected with the same amount of normal saline every 2 days for 3 weeks. According to a previous study [37], approximately 1 × 10^6^ exosomes produced by AFSCs were injected into POF mice via the tail vein every 2 days for 4 weeks, and the same amount of normal saline was injected into the tail vein of the POF group. All animal experiments are conducted according to the guidelines of the National Institutes of Health on the care and use of laboratory animals. The research program was also approved by the Ethics Committee of the Experimental Animal Center of the General Hospital affiliated with Shanghai Jiao Tong University (2020AW126).

### 2.3. Flow Cytometry Screening and *In Vitro* Expansion of CD44+/CD105+ Amniotic Fluid Stem Cells (AFSCs)

Amniocentesis was performed to collect amniotic fluid cells from women at 18–22 weeks of pregnancy under the guidance of an ultrasound. The cells were then incubated for 30 min with FITC-anti-CD44 and PE-anti-CD105 antibodies (at a concentration of 0.5 *μ*g/10^5^ cells) at 4°C. After washing with PBS (Sigma-Aldrich) containing 0.1% bovine serum albumin (BSA) three times, the amniotic fluid cells were resuspended in 0.5 mL of PBS-BSA and sorted (50) by flow cytometry (FACSCalibur; BD Biosciences, San Jose, CA, USA) to obtain purified CD44+/CD105+ AFSCs. The subgroup cells were inoculated into DMEM: F12 (1 : 1) medium (Gibco; 1 × 10^6^ cells/mL) supplemented with 10 ng/mL bFGF, 10 ng/mL EGF (both from Sigma-Aldrich), 10% fetal bovine serum, and 2 mM L-glutamine (both from Gibco). The cells were cultured at 37°C and 5% CO_2_ to 80% density. All cells were passaged four times under the same conditions before use in subsequent experiments.

### 2.4. Extraction and Labeling of Exosomes

Exosomes were isolated from the cell culture supernatant according to a previously established ultracentrifugation method [38, 39]. Briefly, the supernatant of CD44+/CD105+ AFSCs was collected and centrifuged at a speed of 500 × *g* for 10 min to remove cell fragments and apoptotic bodies. The supernatant was then centrifuged at a speed of 16,500 × *g* for 20 min to collect microvesicles. The supernatant was passed through a 0.22 *μ*m filter (Merck Millipore Ltd., Tullagreen, Carrigtwohill, Co. Cork, Ireland) to remove proteins and fragments. Finally, the exosomes were precipitated by ultracentrifugation at 118,000 × *g* for 70 min and resuspended in PBS mixed with Dil green dye.

### 2.5. Electron Microscopic Analysis of the Exosomes Derived from CD44+/CD105+ AFSCs

The exosomes derived from AFSCs were diluted to 1 mg/mL with PBS, then placed onto a glow discharge copper grid on a filter paper, and dried under an infrared lamp for 20 min. Then, 3% (*w*/*v*) phosphotungstic acid (Electron Microscopy Sciences, Washington, PA, USA) was added, and the grid was air-dried at room temperature. The exosomes were examined under a transmission electron microscope with an acceleration voltage of 80 kV (Hrel 600; Hitachi, Tokyo, Japan).

### 2.6. Flow Cytometry (FCM) Analysis

Based on our previous studies [5, 6, 36], each group of cells was inoculated into a six-well plate with 3 × 10^5^ cells per well. After incubation with exosomes or PBS (negative control) for seven days, the cells were harvested and resuspended in 1 mL PBS. The cells were then fixed in 70% cold ethanol and stored at 4°C for more than 48 h. Before FCM analysis, the fixed cells were centrifuged, washed twice with PBS, and resuspended in a solution containing 50 *μ*L/mL PI or 50 *μ*L/mL Annexin V and 250 *μ*g/mL RNase A (Sigma-Aldrich). The cell suspension was incubated at 4°C for 30 min and then analyzed by FACS (FCM-500; Beckman Coulter, Pasadena, CA, USA).

### 2.7. Luciferase Report Assay

A luciferase reporter assay was performed as previously described [5]. Briefly, OGCs were seeded in 48-well plates at the concentration of 30,000 cells/well and cotransfected with 400 ng of miR-369-3p oligo RNA or miR-mut oligo RNA (GenePharma, Shanghai, China) and 20 ng psiCHECK-2, psiCHECK-2-YAF2, or psiCHECK-2-YAF2-mut (Novobiosci, Shanghai, China) using Lipofectamine 2000, according to the manufacturer's protocol. Luciferase activity was measured after 48 h using the Dual-Luciferase Reporter Assay System (Promega, Madison, WI, USA).

### 2.8. RNA Extraction and Quantitative Real-Time PCR Analysis

Based on our previous studies [5, 6, 36], total RNA was isolated from cells or tissues using TRIzol reagent (Invitrogen, Life Technologies Corporation, Grand Island, NY, USA) as instructed by the manufacturer. RNA samples were treated with DNase I (Sigma-Aldrich). After quantification, the ReverTra Ace-First Strand cDNA Synthesis kit (Toyobo, Toyobo (Shanghai) Biotech Co. Ltd., Shanghai, China) or the miRcute Enhanced miRNA cDNA First-Strand Synthesis kit (Tiangen Biotech (Beijing) Co. Ltd., Beijing, China) was used to reverse transcribe the cDNA. Then, quantitative real-time PCR was performed using the RealPlex4 real-time PCR detection system (Eppendorf, Hamburg, Germany) and the SYBR Green Realtime PCR Master Mix (Toyobo). The reaction conditions were 40 cycles of denaturation at 95°C and annealing at 58°C for 45 s. The relative quantitative method was used to quantify the target cDNA. The comparative cycle threshold (Ct) method was used to determine the *n*-fold difference relative to the control (calibrator, 18S rRNA), and steady-state mRNA levels were reported relative to the calibrator. For each sample, the Ct value of the marker gene was standardized using the formula ΔCt = Ct_genes − Ct_18S rRNA. To determine the level of phase alignment, the following formula was used: ΔCt = ΔCt_treatment_group − ΔCt_control_group. Three biological repeats were performed for each reaction. The 2˗*ΔΔ* Ct method was used to measure the expression levels of relative markers. The internal control 18S rRNA was used as a standardizing agent (Table S1).

### 2.9. Immunofluorescence Staining

Based on our previous studies [5, 6, 36], all fresh tissues were fixed in 4% paraformaldehyde (Sigma-Aldrich) for 30 min, dehydrated using an ethanol gradient, paraffin-embedded, then sliced (thickness 6 *μ*m), and dewaxed with xylene. Tissue sections were then incubated with immunohistochemical sealing solution (Beyotime Biotechnology Co. Ltd., Zhejiang, China) at 37°C for 30 min, followed by rinsing with an immunohistochemical cleaning solution (Beyotime) for 5 min at room temperature, three times. The sections were then incubated with the primary antibody at 37°C for 45 min. After rinsing with immunohistochemical cleaning solution for 5 min at room temperature, three times, the secondary antibody was added and incubated at 37°C for 45 min. After three further rinses with immunohistochemical cleaning solution, the glass slides were treated with immunofluorescence sealing solution (Sigma-Aldrich) and visualized by fluorescence microscopy (Table S2).

### 2.10. Western Blot Analysis

Based on our previous studies [5, 6, 36], 2 × loading lysis buffer (50 mM Tris-HCl, pH 6.8, 2% sodium dodecyl sulfate, 10% *β*-mercaptoethanol, 10% glycerol, and 0.002 bromophenol blue) was used to cleave cells or tissues, and the total protein extract was separated by 12% sodium dodecyl sulfate- (SDS-) polyacrylamide gel electrophoresis and transferred to polyvinylidene fluoride membrane (EMD Millipore, Billerica, MA, USA). After sealing with 5% (*w*/*v*) skimmed milk powder, the membrane was washed three times (10 min each) with Tris buffered saline containing Tween-20 (TBST; Sigma-Aldrich) at room temperature. The membrane was then incubated with the primary antibody overnight at 4°C. After thorough washing, the membrane was incubated with an appropriate secondary antibody for 1 h. After washing with TBST three times (10 min each) at room temperature, the immunoreactivity was observed with an enhanced ECL kit (PerkinElmer Life Science, Norwalk, CT, USA) (Table S2).

### 2.11. Statistical Analysis

Each experiment was performed at least three times. Data are shown as the mean ± standard error and were analyzed using the Student's *t*-test when appropriate. Differences were considered significant at *P* < 0.05.

## 3. Results

### 3.1. Differences in the MicroRNA Carried by Exosomes Derived from Amniotic Fluid Stem Cells (AFSCs) and Exosomes Derived from Nonstem Cells

**Figure 1:**
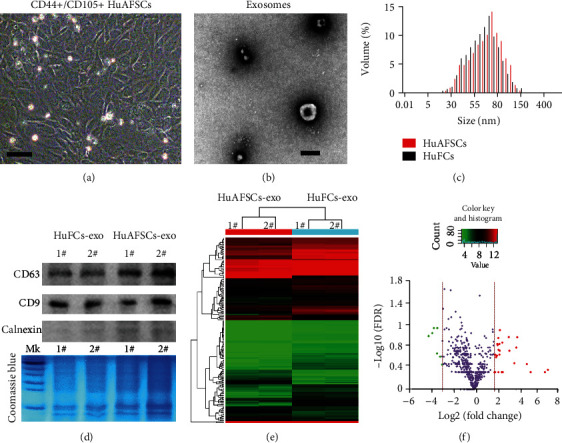
Differences in the microRNA carried by exosomes derived from amniotic fluid stem cells (AFSCs) and exosomes derived from nonstem cells. (a) Morphology of the CD44+/CD105+ subpopulation isolated from human primary amniotic fluid. Original magnification, 200x. (b) Phenotype of HuAFSC-exosomes detected by electron microscopy. Scale bar: 100 nm. (c) The size of HuAFSC-exosomes and HuFCs-exosomes was assessed by nanoFCM. (d) Western blot analysis of exosome marker expression in HuAFSC-exosomes and HuFCs-exosomes. (e) miRNA-Seq results revealed several genes that showed different expression levels between the HuAFSC-exosomes and HuFCs-exosomes. (f) Volcano plot of significant differentially expressed miRNA with a fold-change [log2(HuAFSCs/HuFCs)] > −2 or < ˗3, *P* < 0.05; green and red colors represent downregulated and upregulated expressions, respectively.

According to our previous reports [12, 13], we sorted and enriched CD44+/CD105 + -derived stem cells from human amniotic fluid by FCM (Figure 1(a)). Transmission electron microscopy showed that exosomes were released into the medium during *in vitro* culture (Figure 1(b)). Particle size detection confirmed that the exosomes were 30–150 nm in diameter (Figure 1(c)). Western blot analysis showed that both CD44+/CD105+ HuAFSCs and CD44˗/CD105˗ HuFCs could release exosomes, and the exosomes enriched by ultracentrifugation all reached the standards of CD63 and CD9 but did not express calnexin (Figure 1(d)). The flow cytometric results demonstrated the positive expression of stemness markers CD29, CD90, and CD105 (figure S1A), and differentiation assays showed that HuAFSCs had osteogenic, adipogenic, and chondrogenic abilities (figure S1B).

**Figure 2:**
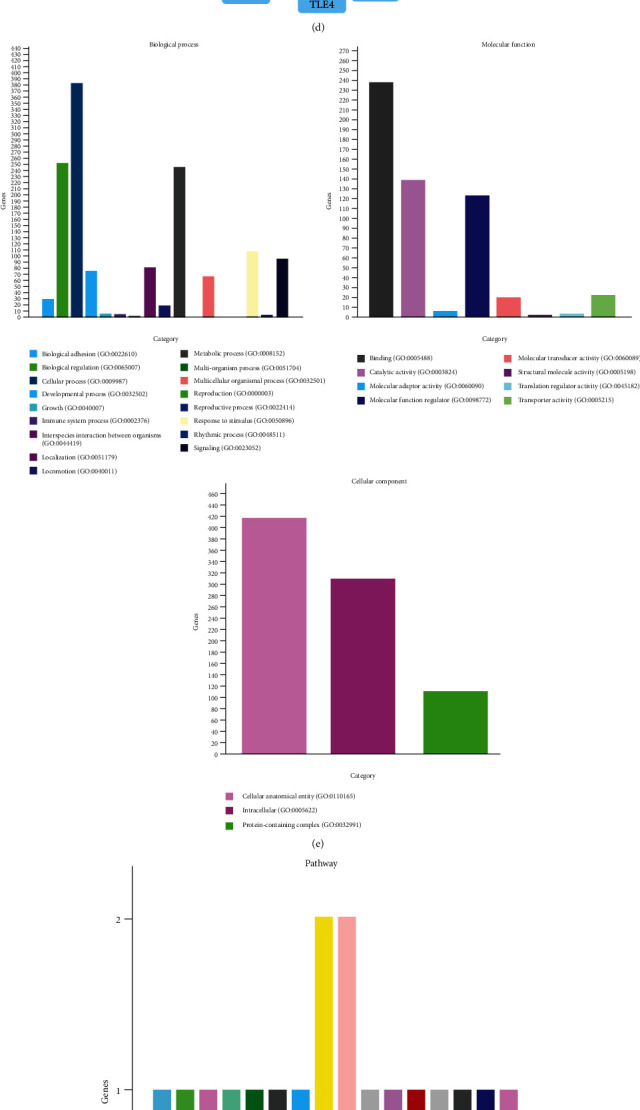
miR-369-3p is a crucial miRNA. (a) Top differentially expressed or crucial miRNAs are listed. (b) qPCR validation of differentially expressed key miRNAs (^∗^*P* < 0.05 vs. HuFCs-exosomes; ^∗∗^*P* < 0.01 vs. HuFCs-exosomes; *t*-test; *n* = 4). (c) The intersection of the miRNA-Seq results and the qPCR results. (d) Target predictions indicated more than one target for miR-369-3p (total context ++ score ≤ −0.26). (e) GO analysis showed that the target genes of miR-369-3p were most significant in the terms “cellular process,” “molecular interaction,” and “cellular anatomical entities.” (f) KEGG analysis showed that the genes were involved in the gonadotropin-releasing hormone receptor pathway and the Huntington disease pathway.

RNA-Seq was used to analyze the specific expression of microRNAs in CD44+/CD105+ HuAFSC-exosomes. Under the conditions of fold-change [log_2_(HuAFSCs/HuFCs)] > −2 or < ˗3, *P* < 0.05, six miRNAs (*hsa-miR-196a-5p*, *hsa-miR-589-5p*, *hsa-miR-576-3p*, *hsa-miR-296-3p*, *hsa-miR-369-3p*, and *hsa-miR-204-3p*) showed significantly increased expression in CD44+/CD105+ HuAFSC-exosomes, and four miRNAs (*hsa-miR-200a-5p*, *hsa-miR-200c-3p*, *hsa-miR-3615*, and *hsa-miR-125b-1-3p*) showed decreased expression (Figure 2(a)). Subsequently, qPCR confirmed only three miRNAs (*hsa-miR-200a-5p*, *hsa-miR-3615*, *hsa-miR-369-3p*) showed differential expression (Figure 2(b)). Of these three miRNAs, only *hsa-miR-369-3p* showed increased expression (Figure 2(c)). Prediction of the target genes of *miR-369-3p* by TargetScan (http://www.targetscan.org/vert_80/) revealed approximately 48 candidate target genes (total context ++ score ≤ −0.26) (Figure 2(d)). Gene ontology (GO) analysis showed that the biological processes of the differentially expressed miRNA target genes were mainly concentrated to “cellular process,” “biological regulation,” and “metabolic process,” and the molecular functions were mainly concentrated to “molecular interaction,” “catalytic activity,” and “molecular functional regulation,” and cells mainly comprised “cellular anatomical entities” and “intracellular and protein complexes” (Figure 2(e)). Kyoto Encyclopedia of Genes and Genomes (KEGG) analysis showed that differentially expressed miRNA target genes were mainly concentrated into the “gonadotropin-releasing hormone receptor pathway” and the “Huntington disease pathway” (Figure 2(f)).

### 3.2. Mouse *YAF2* Gene Is a Potential Target of Human miR-369-3p

**Figure 3:**
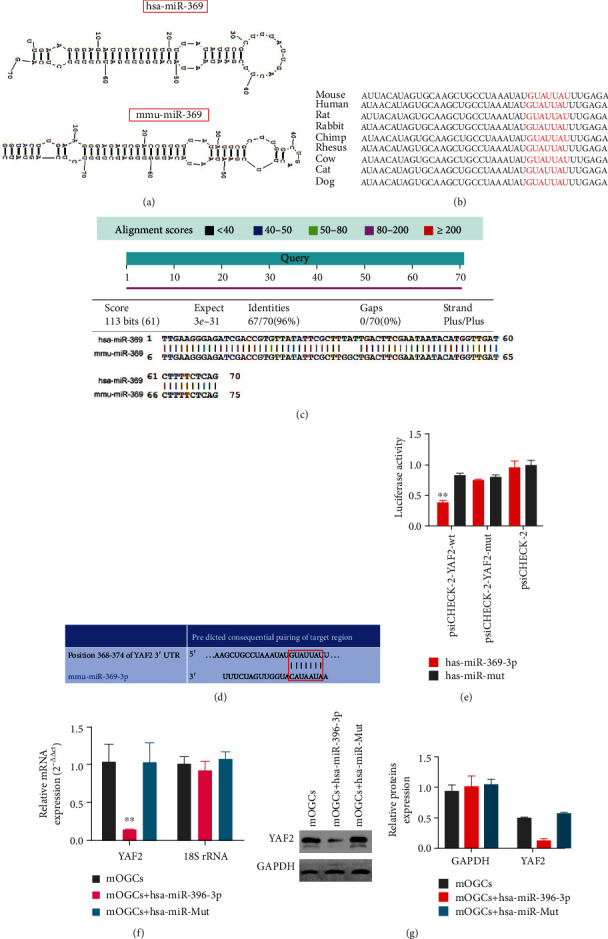
Mouse *YAF2* gene is a potential target of human miR-369-3p. (a) The base structures appear similar between hsa-miR-369 and mmu-miR-369 (National Center for Biotechnology Information, BLAST). (b) The mature miR-369-3p sequence was completely conserved among different species. (c) hsa-miR-369-3p and mmu-miR-369-3p shared 96% nucleotide homology. (d) mmu-miR-369 was completely reverse complementary to the 3′-UTR of the *YAF2* gene (TargetScan, http://www.targetscan.org/vert_80/). (e) The luciferase reporter assay results showed that when the luciferase-encoding gene carried the YAF2 3′-UTR, the overexpression of hsa-miR-369-3p significantly reduced the expression of luciferase activity (^∗∗^*P* < 0.01 vs. hsa-miR-mut group; *t*-test; *n* = 4). (f) The qRT-PCR assay results showed that in mOGCs of the hsa-miR-369-3p overexpressing group, the YAF2 mRNA expression levels were significantly lower than those in the hsa-miR-mut overexpressing group (^∗∗^*P* < 0.01 vs. hsa-miR-mut group; *t*-test; *n* = 4). (g) The western blot results showed that in mOGCs of the hsa-miR-369-3p overexpressing group, the YAF2 expression levels were significantly lower than those in the has-miR-mut overexpressing group (^∗∗^*P* < 0.01 vs. hsa-miR-mut group; *t*-test; *n* = 3).

It was not clear whether *hsa-miR-369-3p* released by HuAFSCs could target mouse genes. Therefore, we used BLAST (National Center for Biotechnology Information) to analyze the secondary structure of *miR-369-3p* in humans and mice and found that it was highly similar (Figure 3(a)); *hsa-miR-369-3p* and *mmu-miR-369-3p* also shared 96% nucleotide homology (Figure 3(c)). We also found that the mature *miR-369-3p* sequence was completely conserved among different species (Figure 3(b)). These results suggested that human *miR-369-3p* may directly regulate mouse genes. Next, we used the TargetScan (http://www.targetscan.org/vert_80/) tool to detect the target gene of *mmu-miR-369-3P* and found that it was completely reverse complementary to the 3′-UTR of the *YAF2* gene (Figure 3(d)). A luciferase reporter assay using the specific sequence of the 3′-UTR of the *YAF2* gene showed that overexpression of human *miR-369-3p* in mouse OGCs significantly inhibited luciferase activity (Figure 3(e)). In addition, the results of qPCR and western blotting showed that compared with the overexpression of miR-mut in mouse OGCs, the expression of endogenous *YAF2* decreased significantly after overexpression of *miR-369-3p* in mouse OGCs *in vitro* (Figures 3(f) and 3(g)). These findings confirmed that *miR-369-3p* could target and silence *YAF2* expression.

### 3.3. The Therapeutic Effect of HuAFSC-Exosomes on Ovarian Granulosa Cells (OGCs), as Demonstrated by Inhibiting the Cellular Expression of YAF2/PDCD5/p53

**Figure 4:**
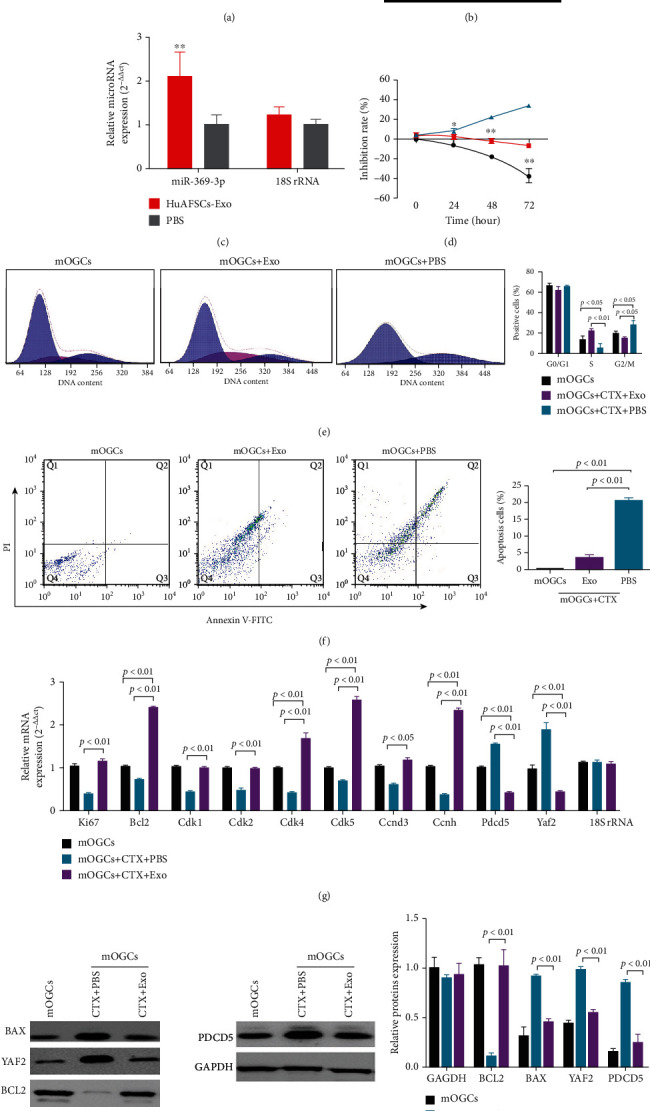
Therapeutic effect of HuAFSC-exosomes on ovarian granulosa cells (OGCs), as demonstrated by inhibiting the cellular expression of YAF2/PDCD5/p53. (a) CTX-damaged OGCs were treated with HuAFSC-exosomes. (b) The Dil-labelled HuAFSC-exosomes were detected in OGCs. (c) The qRT-PCR assay results showed that the expression level of hsa-miR-369-3p in the exosome coculture group was significantly higher than that in the PBS group (^∗∗^*P* < 0.01 vs. PBS group; *t*-test; *n* = 4). (d) The results of MTT detection suggested that HuAFSC-exosomes could significantly alleviate the persistent proliferation inhibition of CTX-damaged mOGCs (^∗^*P* < 0.05 vs. mOGCs-CTX-PBS group; ^∗∗^*P* < 0.01 vs. mOGCs-CTX-PBS group; *t*-test; *n* = 3). (e) FCM assay results showed that HuAFSC-exosomes significantly reduced the number of mOGCs arrested by CTX in the G2max M phase (*P* < 0.05 vs mOGCs-CTX-PBS group; *t*-test; *n* = 3), while increasing the number of mOGCs in the S phase (*P* < 0.01 vs. mOGCs-CTX-PBS group; *t*-test; *n* = 3). (f) Annexin V-FITC staining/FCM assay results showed that HuAFSC-exosomes significantly inhibited apoptosis in mOGCs cell lines (*P* < 0.01 vs. mOGCs-CTX-PBS group; *t*-test; *n* = 3). (g) qRT-PCR results showed that the mRNA expression levels of Ki67, Bcl2, CDK1, CDK2, CDK4, CDK5, CCND3, and CCNH were significantly higher in the mOGCs of the HuAFSC-exosomes group than in the OGCs of the PBS group, and the mRNA expression levels of PDCD5 and YAF2 were significantly lower in the mOGCs of the HuAFSC-exosomes group than in the OGCs of the PBS group (*P* < 0.01 vs. mOGCs-CTX-PBS group; *t*-test; *n* = 3). (h) Western blot analysis showed that HuAFSC-exosomes significantly reduced the expression of BAX, YAF2, and PDCD5 in mOGCs induced by CTX and promoted the expression of BCL2 (*P* < 0.01 vs. mOGCs-CTX-PBS group; *t*-test; *n* = 3).

Primary mouse OGCs were isolated, cultured, and then treated with cyclophosphamide (Cytoxan (CTX)). Dil-labelled HuAFSC-exosomes, at a previously reported concentration [40], were added for coculture (Figure 4(a)). After 3 days of coculture, green fluorescence could be seen in mouse (m)OGCs, indicating that the Dil-labelled HuAFSC-exosomes had been phagocytized successfully (Figure 4(b)). Quantitative PCR detection showed that the expression level of *hsa-miR-369-3p* in the exosome coculture group was significantly higher than that in the control group (PBS coculture group) (Figure 4(c)). The results of MTT detection suggested that HuAFSC-exosomes could significantly alleviate the persistent proliferation inhibition of mOGCs induced by CTX (Figure 4(d)). The results of FCM indicated that HuAFSC-exosomes could significantly reduce the number of mOGCs arrested by CTX in the G2max M phase, while increasing the number of mOGCs in the S phase (Figure 4(e)). The results of Annexin V-FITC/PI double staining and FCM detection suggested that HuAFSC-exosomes could significantly reduce mOGC apoptosis induced by CTX (Figure 4(f)).

In addition, qPCR revealed that HuAFSC-exosomes could significantly promote the expression of CTX-induced mOGC core proliferation factor *Ki67*, antiapoptosis gene *Bcl2*, and cell cycle regulatory proteins *CDK1*, *CDK2*, *CDK4*, *CDK5*, *CCND3*, and *CCNH*, while significantly downregulating the expression of *PDCD5* and *YAF2* (Figure 4(g)). Western blot analysis showed that HuAFSC-exosomes could significantly reduce the expression of BAX, YAF2, and PDCD5 in mOGCs induced by CTX and promoted the expression of BCL2 (Figure 4(h)). Taken together, these results confirmed that HuAFSC-exosomes could exert therapeutic effects by inhibiting the expression of hsa-miR-369-3p-targeted YAF2 in OGCs and inhibiting the stability of PDCD5/p53.

### 3.4. HuAFSC-Exosomes Rely on hsa-miR-369-3p to Inhibit the Expression of YAF2 in OGCs to Achieve Therapeutic Effects in POF Mice

**Figure 5:**
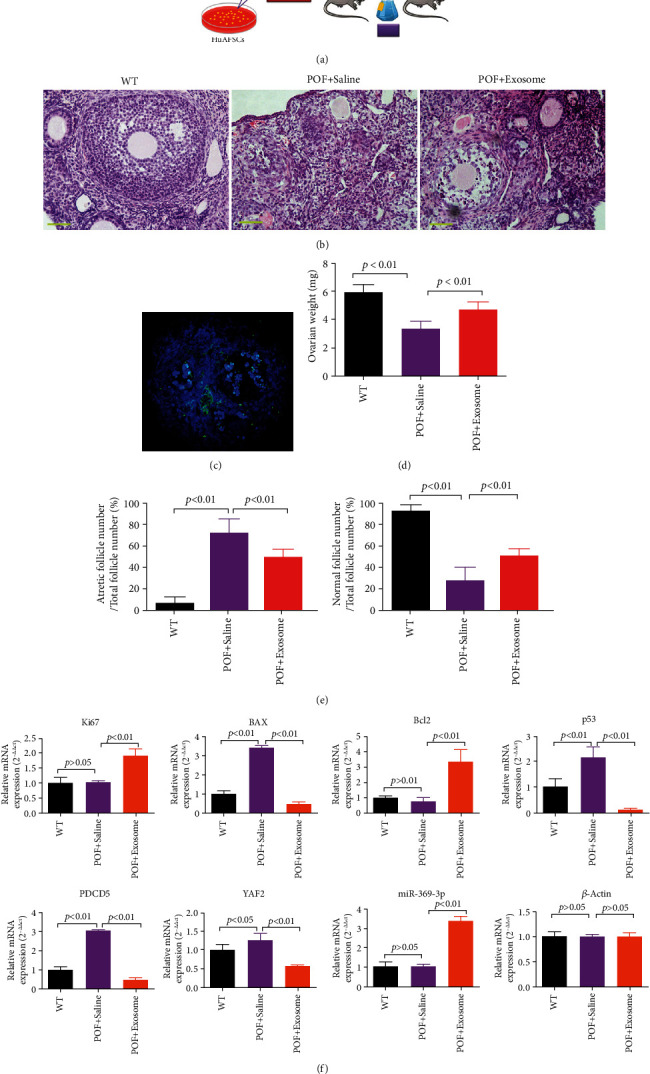
HuAFSC-exosomes rely on hsa-miR-369-3p to inhibit the expression of YAF2 in OGCs to achieve therapeutic effects in POF mice. (a) Experimental design. (b) Ovarian pathological identification was carried out by hematoxylin and eosin staining (magnification 200x). (c) Immunofluorescence analysis showed that the Dil-labelled HuAFSC-exosomes were located in the OGCs of mice. (d) Ovarian weight was higher in the HuAFSC-exosome-treated mice than in the saline-treated mice (*P* < 0.01 vs. POF + saline-treated mice; *t*-test; *n* = 8). (e) Normal follicle count was higher in the HuAFSC-exosome group than in the saline group, and fewer atretic follicles were present in the HuAFSC-exosome-treated mice than in the saline-treated mice (*P* < 0.01 vs. POF + saline-treated mice; *t*-test; *n* = 8). (f) qRT-PCR results showed that the mRNA expression levels of Ki67, Bcl2, and miR-369-3p were significantly higher in the mOGCs of the HuAFSC-exosome-treated mice than in the OGCs of the saline-treated mice, and the mRNA expression levels of BAX, P53, PDCD5, and YAF2 were significantly lower in the mOGCs of the HuAFSC-exosome-treated mice than in the OGCs of the saline-treated mice (*P* < 0.01 vs. POF + saline-treated mice; *t*-test; *n* = 3).

**Figure 6:**
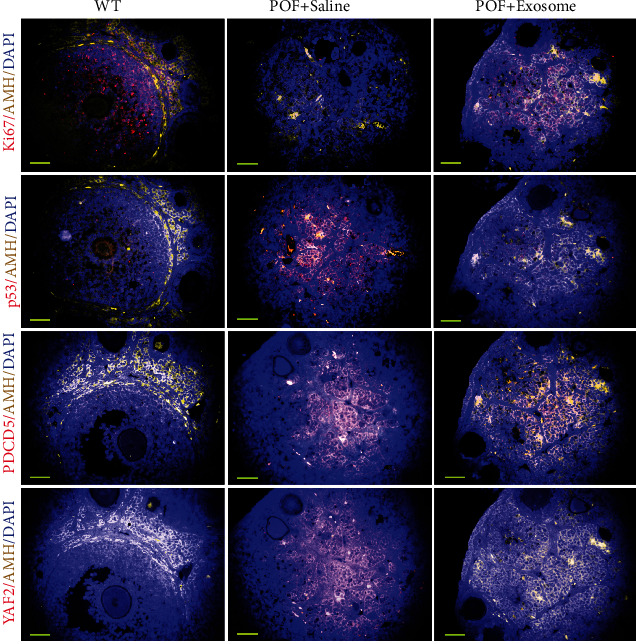
Immunofluorescence staining results for Ki67, P53, PDCD5, and YY2 (magnification 400x).

**Figure 7:**
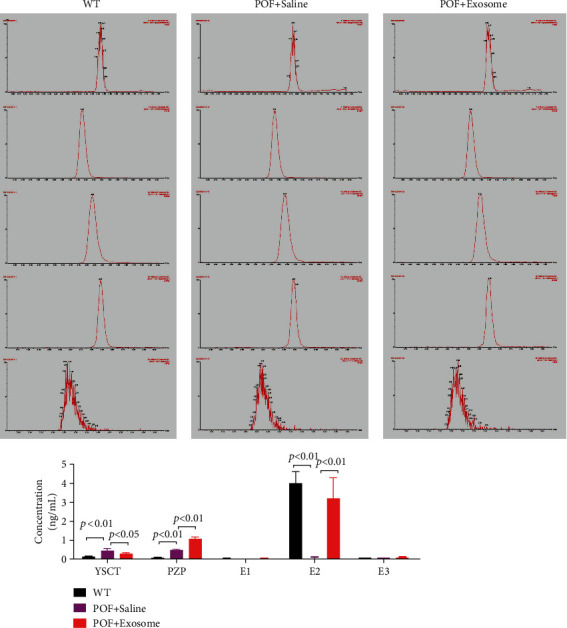
Steroid hormone levels in mice tested by HPLC-MS/MS. YSCT: pregnenolone; PZP: pregnancy zone protein; E1: estrone; E2: estradiol; E3: estriol (*P* < 0.01 vs. POF + saline-treated mice, *t*-test, *n* = 4; *P* < 0.01 vs POF + saline-treated mice, *t*-test, *n* = 4).

When Dil-labelled HuAFSC-exosomes were injected into the tail vein of POF mice (Figure 5(a)), they accumulated in the ovaries (Figure 5(c)). We examined the ovarian tissue of mice using pathological and biochemical methods. Mature follicles appeared in the ovaries of POF mice after HuAFSC-exosome transplantation, and the number of atretic follicles decreased significantly (Figures 5(b) and 5(e)). A considerable increase in the weight of the ovaries was also detected (Figure 5(d)). Quantitative PCR showed increased expression of *miR-369-3p*, *Ki67*, and *Bcl2* and decreased expression of *YAF2*, *PDCD5*, *p53*, and *BAX* in ovarian tissue after HuAFSC-exosome transplantation (Figure 5(f)). Immunofluorescence analysis confirmed that HuAFSC-exosomes significantly increased the expression of Ki67 and decreased the expression of YAF2, PDCD5, and p53 in the ovaries of POF mice (Figure 6). In addition, the results of steroid hormone determination by HPLC-MS/MS showed that the levels of estradiol (E2) and pregnancy band protein (PZP) in the peripheral blood of POF mice injected with HuAFSC-exosomes were significantly higher than those in POF mice injected with PBS, while the level of pregnenolone (YSCT) hormone decreased (Figure 7).

## 4. Discussion

At present, the most commonly used treatment for POF is hormone replacement therapy, which simulates physiological hormone release. However, hormone replacement therapy can induce many side effects, such as neurodegenerative diseases, liver injury, and venous thrombosis, which in turn increases the risk of endometrial cancer, ovarian cancer, and breast cancer [9, 13]. Stem cell transplantation is gaining increased interest as a potential alternative method for the treatment of POF [12, 41, 42]. Because stem cells can differentiate into a variety of adult cells and have the advantage of low immunogenicity, they are considered high-quality seeds for cell therapy [12–14]. However, our previous study found that not all cells among AFSCs exhibit the same phenotype, suggesting that it is not a single cell community [13]. For stem cell therapy, it is vital to purify and enrich stem cell subsets with pluripotent characteristics. Therefore, exosomes derived from stem cells may offer a promising alternative for cell therapy [16–18]. Exosomes carry small peptides, cytokines, nucleic acids, and other substances that can achieve therapeutic effects on host cells or tissues and therefore potentially constitute independent high-quality gene therapy seeds. In this study, exosomes were isolated and enriched from HuAFSC subsets of CD44+/CD105+, and the exosomes were transplanted into a POF model *in vitro* and *in vivo*. Our results confirmed that the exosomes produced by HuAFSCs could achieve therapeutic effects in a mouse POF model. This demonstrated that the exosomes derived from stem cells not only had the same repairability of stem cells but also had the advantage of low immunogenicity.

To investigate the mechanism of action of the human stem cell exosomes, we analyzed the differential expression profiles of miRNAs between AFSC-derived exosomes and amniotic fluid nonstem cell-derived exosomes and found differential expression of several miRNAs. The most highly expressed miRNA in exosomes derived from AFSCs was hsa-miR-369-3p, suggesting a potential key functional role for this miRNA. Through sequence comparisons, we found that the mature miRNA nucleic acid sequence of both human and mouse miR-369-3p was conserved. The binding site sequence to target genes was also conserved between human and mouse miR-369-3p. This finding provided theoretical evidence for the generality of stem cell-derived exosome therapy. We subsequently confirmed that human miR-369-3p could target and silence mouse YAF2 expression. During our study, we found that when CTX was used to establish the OGC injury model and the mouse POF model, the *in vitro* and *in vivo* results suggested that OGCs undergo aging, proliferation inhibition, and death, and cell cycle analysis confirmed that the proportion of S phase cells decreased significantly. Therefore, we speculated that CTX could inhibit cell proliferation and division by activating the cell proliferation inhibition signal pathway. Coincidentally, it has been reported that YAF2, as a binding molecule of YinYang-1 (YY-1) protein, played an important role in transcriptional regulation by stabilizing the PDCD5 structure and promoting p53 to inhibit cell proliferation [32]. As a member of the programmed cell death family, PDCD5 protein plays an important positive regulatory role in promoting apoptosis. It can participate in apoptosis by activating stable caspase-3 [35], p53 [34], HIF-1 *α* [33], and other pathways. In our POF model, OGCs highly expressed p53 and PDCD5. The above experimental results strongly suggested that the YAF2/PDCD5/p53 signal pathway was activated in the process of CTX modeling. When we transplanted the exosomes derived from AFSCs into the POF model, we found that the expression of YAF2, PDCD5, p53, and other proteins in the recipient cells was significantly downregulated, and the symptoms of POF were significantly relieved.

There are still some limitations in this study. First, the role that miR-369-3p plays in AFSCs is limited. Past research has demonstrated that exosome exerted its actions through a multiplicity of mechanisms [19–21]. Further, the mechanisms still need our further exploration. A second limitation is that exosome yield was extremely low, and therefore, exosome production efficiency must be increased. At present, many methods for large-scale production of exosomes were available [43]. These may be worth exploring.

**Figure 8:**
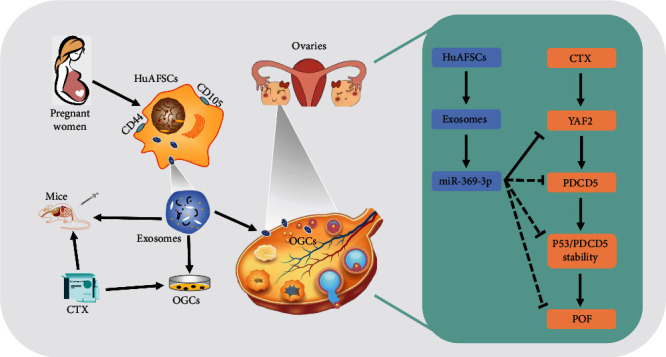
CD44+/CD105+ HuAFSC-exosomes carrying miR-369-3p could specifically downregulate the expression of YAF2, inhibit the stability of PDCD5/p53, and reduce the apoptosis of OGCs, thereby exerting therapeutic effects on POF.

In summary, our findings confirmed that exosomes derived from AFSCs could silence the expression of the YAF2/PDCD5/p53 signal pathway in recipient tissue via the activity of donor miR-369-3p carried by the exosomes, which weakened the activity of the cell proliferation inhibitory signal and exerted therapeutic effects on mouse POF (Figure 8).

## Data Availability

All data are available with the author's consent.

## Supplementary Materials

Supplementary 1 Figure S1: stem cell biomarkers of HuAFSC detected and osteoblast and adipocyte differentiation. (A) The results of FCM detected. (B) The results of cell staining. Magnification: 200x.

Supplementary 2 Table S1: primers for qRT-PCR.

Supplementary 3 Table S2: the list of primary antibodies.
