# Supplementary figures and images for: Human Amniotic Fluid Mesenchymal Stem Cell-Derived Exosomes Inhibit Apoptosis in Ovarian Granulosa Cell via miR-369-3p/YAF2/PDCD5/p53 Pathway
Source: Oxid Med Cell Longev. 2022 Jul 26;2022:3695848. doi: 10.1155/2022/3695848 (PMC9346541; doi:10.1155/2022/3695848)

**A.**

CD29

CD90

CD105

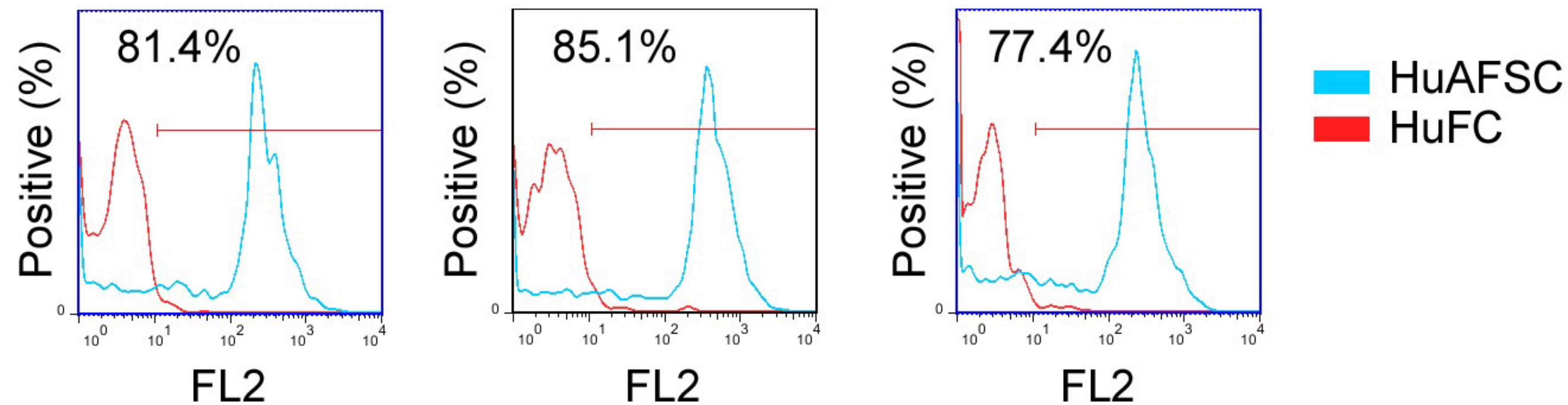**B.**

Alizarin Red S

Von Kossa

Oil Red O

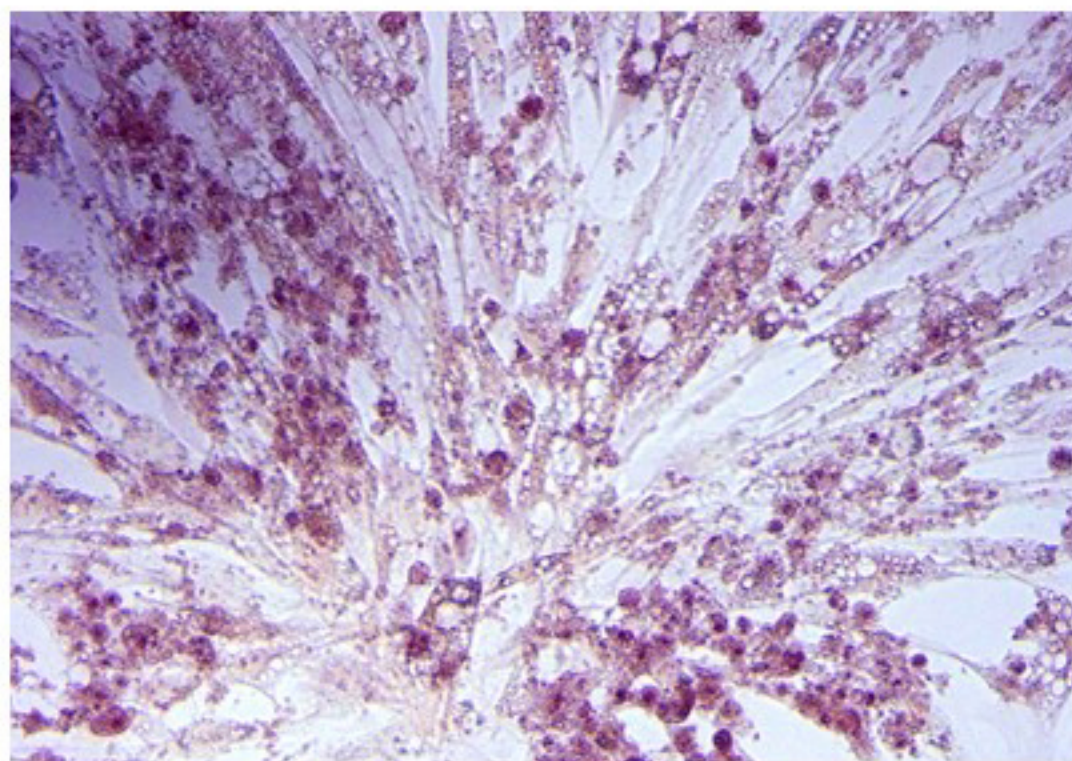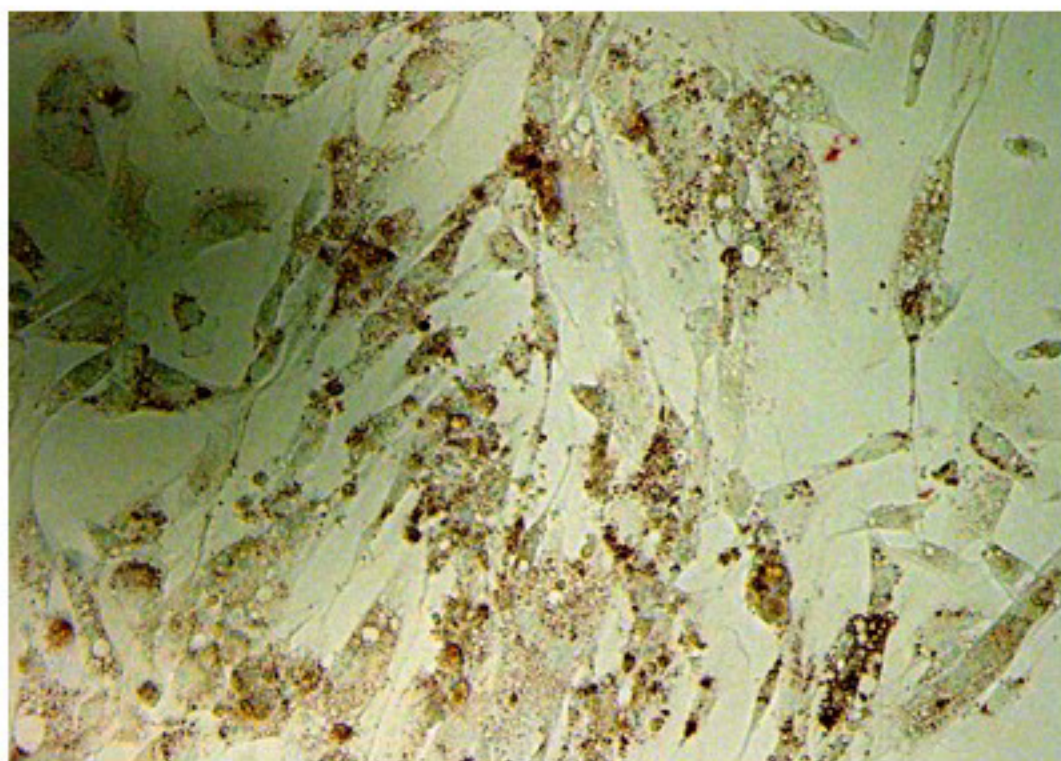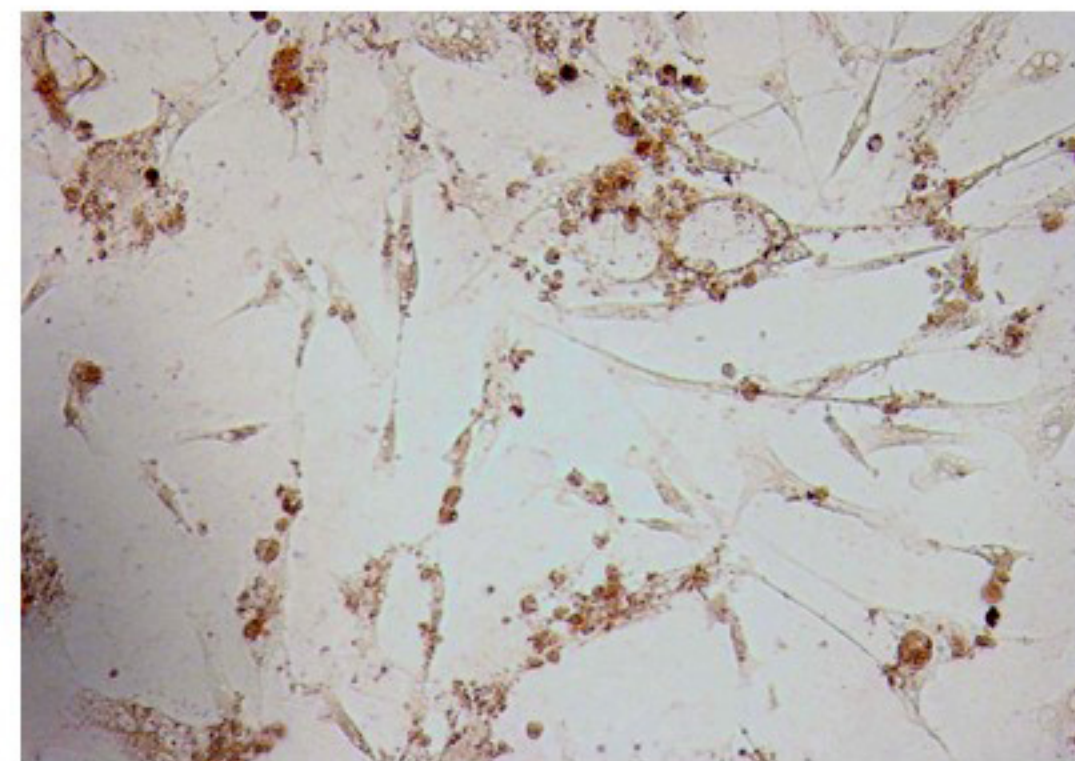

Supplement: Supplementary 1 — Figure S1: stem cell biomarkers of HuAFSC detected and osteoblast and adipocyte differentiation. (A) The results of FCM detected. (B) The results of cell staining. Magnification: 200x. [file 3695848.f1.pdf]
