# Supplementary material for: Human Amniotic Fluid Mesenchymal Stem Cell-Derived Exosomes Inhibit Apoptosis in Ovarian Granulosa Cell via miR-369-3p/YAF2/PDCD5/p53 Pathway
Source: Oxid Med Cell Longev. 2022 Jul 26;2022:3695848. doi: 10.1155/2022/3695848 (PMC9346541; doi:10.1155/2022/3695848)
Supplement: Supplementary 2 — Table S1: primers for qRT-PCR. [file 3695848.f2.docx]

| **Table S1 Primers for qRT-PCR** | |
| --- | --- |
| **Gene** | **Forward (F) and reverse (R) primers (5’→3’)** |
| m18S-FP | AGGGGAGAGCGGGTAAGAGA |
| m18S-RP | GGACAGGACTAGGCGGAACA |
| mYAF2-FP | CCCCCTACCCAGTCGAAGAAA |
| mYAF2-RP | GCGCTACTCCGATCCACATATT |
| mKi67-FP | ATCATTGACCGCTCCTTTAGGT |
| mKi67-RP | GCTCGCCTTGATGGTTCCT |
| BCL2-FP | GAGAGCGTCAACAGGGAGATG |
| BCL2-RP | CCAGCCTCCGTTATCCTGGA |
| CDK1-FP | AGAAGGTACTTACGGTGTGGT |
| CDK1-RP | GAGAGATTTCCCGAATTGCAGT |
| CDK2-FP | CTCTCACGGGCATTCCTCTTC |
| CDK2-RP | CCCTCTGCATTGATAAGCAGG |
| CDK4-FP | AAGGTCACCCTAGTGTTTGAGC |
| CDK4-RP | CCGCTTAGAAACTGACGCATTAG |
| CDK5-FP | CCCTGAGATTGTGAAGTCATTCC |
| CDK5-RP | CCAATTTCAACTCCCCATTCCT |
| CCND3-FP | CGAGCCTCCTACTTCCAGTG |
| CCND3-RP | GGACAGGTAGCGATCCAGGT |
| CCNH-FP | ATCTTCGAGAGAGTCCTCTTGG |
| CCNH-RP | TGGTCTGTATGGATTGTGGACA |
| PDCD5-FP | ATGGCGGACGAAGAACTTGAG |
| PDCD5-RP | GGGCTGACTGATCCAGAACT |
| P53-FP | CTCTCCCCCGCAAAAGAAAAA |
| P53-RP | CGGAACATCTCGAAGCGTTTA |
| BAX-FP | AGACAGGGGCCTTTTTGCTAC |
| BAX-RP | AATTCGCCGGAGACACTCG |
| β-actin-FP | GGCTGTATTCCCCTCCATCG |
| β-actin-RP | CCAGTTGGTAACAATGCCATGT |
