# Supplementary material for: Human Amniotic Fluid Mesenchymal Stem Cell-Derived Exosomes Inhibit Apoptosis in Ovarian Granulosa Cell via miR-369-3p/YAF2/PDCD5/p53 Pathway
Source: Oxid Med Cell Longev. 2022 Jul 26;2022:3695848. doi: 10.1155/2022/3695848 (PMC9346541; doi:10.1155/2022/3695848)
Supplement: Supplementary 3 — Table S2: the list of primary antibodies. [file 3695848.f3.docx]

| **Table S2 List of antibodies** | | |
| --- | --- | --- |
| **Antibodies** | **Companies** | **Applications** |
| Rabbit anti-GAPDH antibody [EPR16891] (ab181602) | Abcam, MA, USA | WB (1:1000) |
| Rabbit anti-CD63 antibody (ab68418) | Abcam, MA, USA | WB (1:1000) |
| Rabbit anti-CD9 antibody (ab223052) | Abcam, MA, USA | WB (1:1000) |
| Rabbit anti-Calnexin antibody (ab227310) | Abcam, MA, USA | WB (1:1000) |
| Rabbit Anti-Bcl-2 antibody [E17] (ab32124) | Abcam, MA, USA | WB (1:1000) |
| Rabbit anti-Bax antibody [EPR18283] (ab182733) | Abcam, MA, USA | WB (1:1000) |
| Rabbit anti-YAF2 antibody [EPR11020] (ab177945) | Abcam, MA, USA | WB (1:1000)  IF (1:300) |
| Rabbit anti-PDCD5 antibody (ab83958) | Abcam, MA, USA | WB (1:1000) |
| Rabbit anti-PDCD5 antibody (ab75430) | Abcam, MA, USA | IF (1:300) |
| Rabbit anti-Ki67 antibody (ab15580) | Abcam, MA, USA | IF (1:300) |
| Rabbit anti-p53 antibody [E26] (ab32389) | Abcam, MA, USA | IF (1:300) |
| Rabbit anti-AMH antibody (ab229212) | Abcam, MA, USA | IF (1:300) |
| Goat anti-Rabbit IgG H&L (HRP) (ab97051) | Abcam, MA, USA | WB (1:1000) |
| Goat Anti-Rabbit IgG H&L (Alexa Fluor® 488) (ab150077) | Abcam, MA, USA | IF (1:300) |
